# Early exposure to environment sounds and the development of cortical auditory evoked potentials of preterm infants during the first 3 months of life

**DOI:** 10.1186/s13104-020-05129-8

**Published:** 2020-06-26

**Authors:** Hannalice Gottschalck Cavalcanti, Aryelly Dayane da Silva Nunes, Brenda Karla Silva da Cunha, Kátia de Freitas Alvarenga, Sheila Andreoli Balen, Antonio Pereira

**Affiliations:** 1 Department of Speech and Language Pathology, Federal University of Paraíba, Cidade Universitária, S/N - Conj. Pres. Castelo Branco III, João Pessoa, PB 58051-900 Brazil; 2 Speech and Language Pathology Graduate Program, Federal University of Rio Grande do Norte, Rua Gen. Gustavo Cordeiro de Farias, S/N, Natal, RN 59012-570 Brazil; 3 Department of Speech and Language Pathology, University of São Paulo, Alameda Dr. Octávio Pinheiro Brisolla, 9-75, Bauru, SP 17012-901 Brazil; 4 Department of Speech and Language Pathology, Federal University of Rio Grande do Norte, Rua Gen. Gustavo Cordeiro de Farias, S/N, Natal, RN 59012-570 Brazil; 5 Department of Electrical and Biomedical Engineering, Institute of Technology, Federal University of Pará, Rua Augusto Correa, S/N, Belém, PA 66075-110 Brazil

**Keywords:** Preterm, Newborn, Brain development, Auditory, Cortex

## Abstract

**Objective:**

Preterm infants are exposed earlier than their term counterparts to unattenuated sounds from the external environment during the sensitive period of the organization of the auditory cortical circuitry. In the current study, we investigate the effect of preterm birth on the course of development of auditory cortical areas by evaluating how gestational age (GA) correlates with the latency of the P1 component of the cortical auditory evoked potential (CAEP) of two experimental groups measured at 1 or 3 months of age.

**Results:**

Our sample consisted of 23 infants delivered at GA ranging from 31.28 to 41.42 weeks and separated into two groups evaluated transversally at 1 or 3 months of corrected age (CA). In the group evaluated at 1-month CA, the latency of the component P1 was similar in both terms and infants classified as late-preterm (GA > 32 weeks). However, in the group evaluated at 3 months CA, P1 latency was significantly smaller in preterms. These preliminary results suggest an acceleration of the development of auditory cortical pathways in preterms, probably due to their early exposure to socially relevant auditory stimuli from the external environment.

## Introduction

The remarkable adaptability of the human brain results from the interplay of both evolutionary and developmental mechanisms. Genetically determined developmental programs set up the stage upon which cortical circuits are sculpted by experience and learning, particularly during the sensitive periods of synaptic plasticity [1–3]. The primary auditory cortex is structurally mature as early as 28 weeks of gestation [4] and already responds electrically to sounds in the low-frequency range associated with speech [5, 6]. This could explain why newborns can discriminate their mothers’ voice immediately after birth [7, 8].

While our understanding of the prenatal emergence of the cortical organization has traditionally depended on animal-based research, due to ethical concerns, preterm birth offers a unique opportunity to investigate this issue in human subjects as well. The extra period of sensory stimulation afforded by preterms allows the earlier maturation of binocular vision and thalamocortical connectivity to the primary somatosensory cortex [9, 10].

Preterm birth is characterized by delivery before 37 weeks of GA [11, 12] and the following sub-categories of preterms are proposed, based on GA: extremely preterm (< 28 weeks), very preterm (28 to < 32 weeks), and moderate to late preterm (32 to < 37 weeks) [13]. Most preterm births (> 70%) are considered late-preterm (34 to < 37 weeks) [14] and they account for about 85 of all births [15]. Since late-preterm births occur during the period when cortical volume increases by 50% (34–40 weeks), there is a pressing need to understand the possible effects of premature exposure to the extrauterine environment in this population [15, 16].

The cortical auditory evoked potential (CAEP) is characterized by waveforms with positive and negative deflections occurring between 0 and 300 ms after sound onset in adults. The earliest components are called P1 and N1 and are already present in newborns [17, 18]. The latency of the P1 component shows a steady decrease until it stabilizes in adulthood [19–22] and has been proposed as a biomarker for the maturation of cortical sensory pathways [17, 21, 23]. In the present study, we evaluate the development of the cortical auditory pathway of late preterms in the first 3 months of life.

## Main text

### Methods

#### Subjects

This study was approved by the Research Ethics Committee of the Federal University of Rio Grande do Norte (#340.110) and written informed parental consent was obtained on behalf of all participants. The participants were 23 newborns (GA: 31.28–41.42 weeks) recruited at the Maternity School of the Federal University of Rio Grande do Norte according to the following inclusion criteria: no signs of hearing problems during routine maternity screening, normal Auditory Brainstem Responses (ABR) with a click and normal threshold for ABR tone burst at 500 Hz, 1000 Hz and 4000 Hz in both ears, normal Distortion Product Otoacoustic Emissions (DPOAE) and tympanometry result with curve type *A* in both ears. Table 1 shows the demographic characteristics and birth outcomes of mothers and infants.

**Table 1:** Demographic characteristics and birth outcomes

	Total sample, *N *= 23	Preterm, *n *= 12	Term, *n *= 11	*p* value
	*n* (%)			
Maternal age^a^				
< 34 (y. o.)^b^	8 (66.67)	2 (66.67)	6 (66.67)	> 0.05
> 35 (y. o.)	4 (33.33)	1 (33.33)	3 (33.33)	
Education				
Less than middle-school	17 (94.44)	8 (100.00)	9 (90.00)	> 0.05
More than middle-school^c^	1 (5.56)	0 (0.00)	1 (10.00)	
Sex of child				
Male	10 (43.48)	6 (46.16)	4 (40.00)	> 0.05
Female	13 (56.52)	7 (53.84)	6 (60.00)	
Gestational age				
31 to < 37 weeks	13 (56.52)	13 (100.0)	0 (0.00)	< 0.05
> 37 weeks	10 (43.48)	0 (0.00)	10 (100.0)	
Birth weight^e^				
< 2500 g	10 (50.00)	10 (83.33)	0 (0.00)	< 0.05
> 2500 g	10 (50.00)	2 (16.67)	8 (100.00)	
Family income (in minimum wages)^f^				
< 1	8 (50.00)	3 (42.85)	5 (55.55)	> 0.05
1 to 5	8 (50.00)	4 (57.15)	4 (44.45)	
Socioeconomic status^g,h^				
A, B, and C	6 (30.00)	1 (9.10)	5 (55.55)	< 0.05
D and E	14 (70.00)	10 (90.90)	4 (44.45)	
NICU admission^i^				
No	6 (42.85)	3 (50.00)	3 (37.50)	> 0.05
Yes^j^	8 (57.15)	3 (50.00)	5 (62.50)	

^a^11 missing values

^b^y.o.: years old

^c^5 missing values

^d^31.28 weeks

^e^3 missing values

^f^8 missing values

^g^3 missing values

^h^Brazil Economic Classification Criteria (https://www.abep.org/criterio-brasil)

^i^9 missing values; ^j^All infants stayed in NICU for 2 days

#### Procedure and stimuli

The design of the study was cross-sectional and the subjects were evaluated at 1 or 3 months after birth and were divided into two groups according to their GA: preterm or term. For the CAEP recordings, subjects were accommodated either on a car seat or in the caregivers’ lap within a sound-attenuated room. All tests were performed while infants were in stage 4 of the Neonatal Behavioral Assessment Scale [24]: alert, awake state.

We used ER-3A insert phones (Etymotic Research, Inc.) for sound delivery to the right ear and the CAEP recordings were performed with a Smart EP USB Jr system with two channels (Intelligent Hearing Systems, Inc.). The CAEP was recorded on channel A, while channel B was used to register eye movements for off-line artifact removal and to determine the rejection level for each session. Disposable surface electrodes were used for the recording procedures. The CAEP was recorded at the midline (Cz) and referenced to the right mastoid. The ground electrode was placed at the left mastoid. All electrode impedances were less than 3 kΩ. A minimum of 150 stimuli was presented and the resulting signal, within an analysis window of − 100 ms pre-stimulus and 500 ms post-stimulus, was averaged at both 70 dB NA and 0 dB NA after band-pass filtering from 1 to 30 Hz. The gain in both channels was 100,000. The rate of the stimulus was 1.9 s.

Auditory responses were recorded in response to a/da/speech stimulus with an intensity of 70 dB HL and with an interstimulus interval of 526.00 ms. The/da/sound was recorded with the software praat (https://www.praat.org) using a unidirectional microphone in an acoustically isolated room [25]. The latency of component P1 was determined as the first positive peak after 50 ms, following a negative decline. The latency of the P1 component was confirmed independently by two experienced judges.

#### Statistical analysis

Only 2-sided tests and nonparametric statistical tests were used due to the non-normal distribution of variables and/or sample size. Sample characteristics were compared using Pearson’s Chi square test or Fisher’s exact test. Results are expressed as mean ± standard deviation. Samples’ comparison at 1- and 3-months CA were performed with a two-tailed Mann–Whitney rank sum U test. The relationship between GA and p1 latency was assessed with the Spearman correlation (*r*). The significance level was set at 0.05.

### Results

**Fig. 1:**
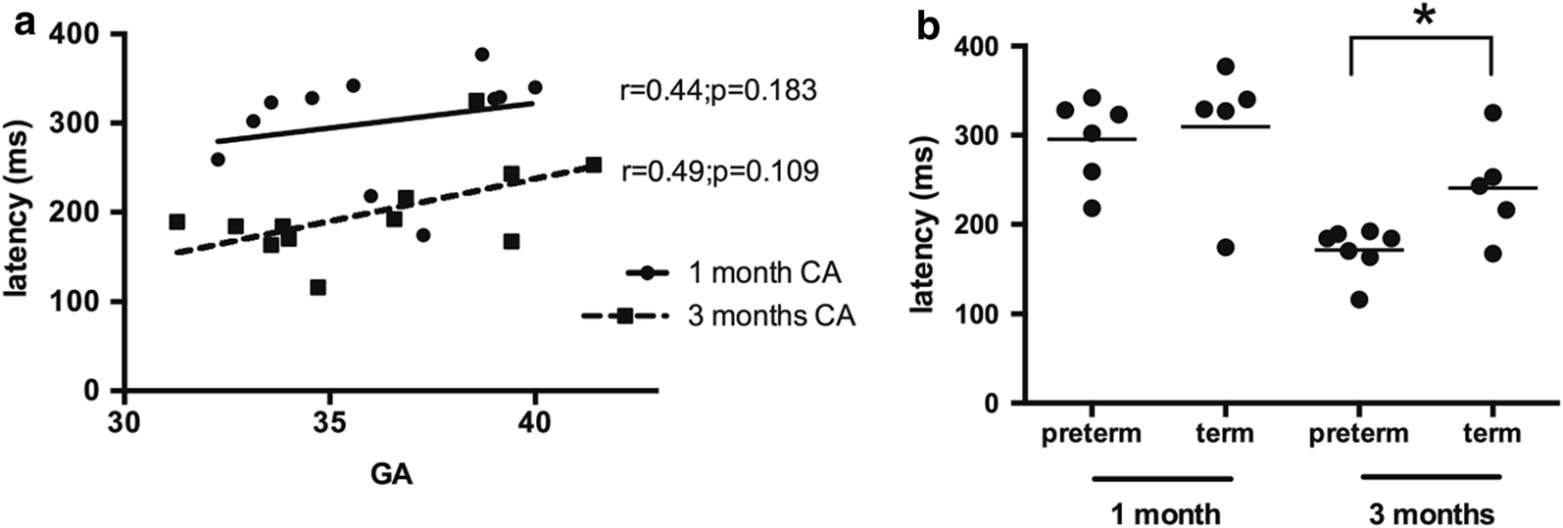
**a** P1 latencies recorded at 1- and 3-months CA as a function of GA. Linear regression lines are superimposed on the raw data for the CAEP recordings at 1- or 3-months CA, respectively. **b** Latency of the P1 component of the CAEP of preterm (GA < 37 weeks) and term (GA ≥ 37 weeks) infants recorded at 1- or 3-months CA. Lines represent the median. *p < 0.05

P1 latency is not correlated with GA at both 1 (*r*_*s*_ = 0.44, *p* = 0.183) and 3 (*r*_*s*_ = 0.49, *p* = 0.109) months CA. We computed linear regression lines to fit the P1 latency data (Fig. 1a) and though the slopes of regression lines were not significantly different (*p *= 0.63) (see Additional file 1: Table S1), their elevations are significantly different from each other (*p* < 0.001) (Fig. 1).

According to Table 1, the demographic characteristics and birth outcomes of terms and preterms are similar, except for birth weight, which is lower in preterms (*p* < 0.05). Our sample is composed mostly of moderate to late preterms (GA 32 to < 37 weeks), which represent about 10% of all births [26]. Their prematurity ranged from 0.15 to 5.72 weeks (average 2.78 ± 1.58 weeks).

**Fig. 2:**
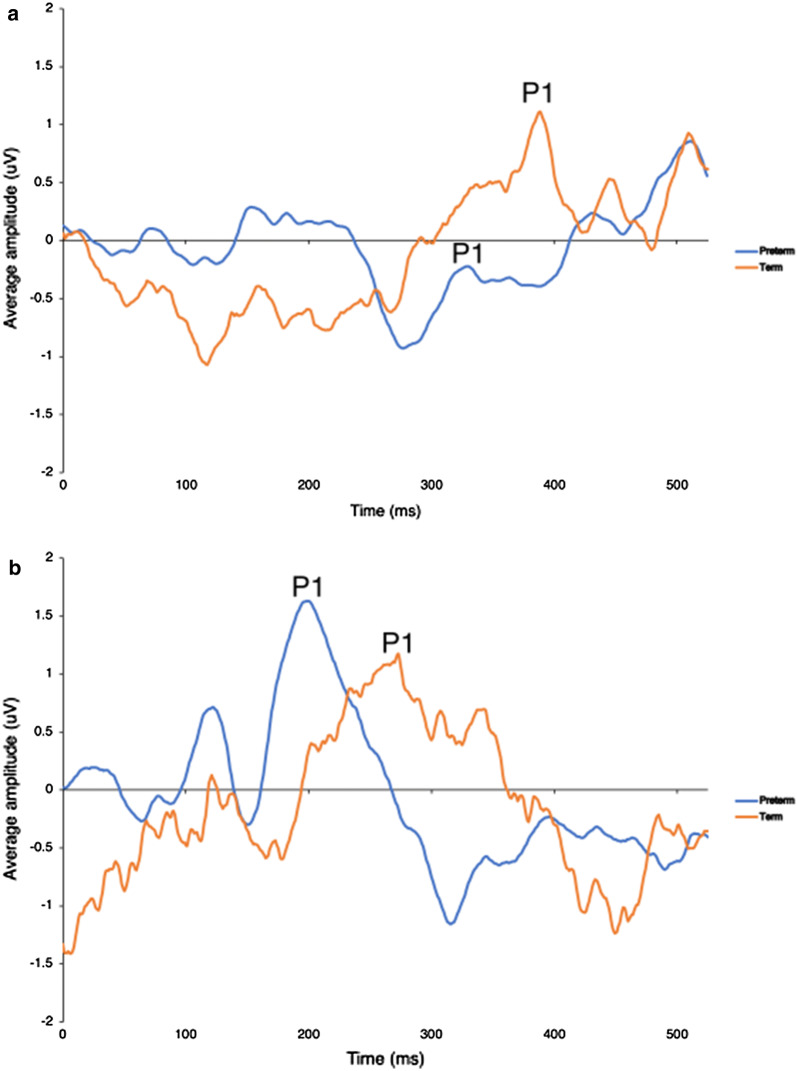
Grand average waveforms of the CAEP of the term and preterm groups recorded at 1- (**a**) and 3-months (**b**) CA. The arrows indicate the P1 component

For the infants evaluated at 1-month, the average latency of the P1 component was not significantly different between terms (309.40 ± 78.32 ms) and preterms (295.30 ± 47.66 ms) (*U* = 10, *p* = 0.4242) (Fig. 1b). However, for the group evaluated at 3-months, average P1 latency was 240.80 ± 57.67 ms for terms and 171.1 ± 26.44 ms for preterms, respectively (Fig. 1b), and significantly lower for the latter (*U *= 5, *p *< 0.05). The grand average CAEP waveforms at 1- and 3-months are shown in Fig. 2a, b, respectively. The latency of the P1 component at 3-months is smaller than at 1-month for the preterm group (176.00 ± 29.16 ms vs 295 ± 47.65 ms; *U* = 1, *p* = 0.005), but not the term group (247.00 ± 64,64 ms vs. 309.40 ± 78.31 ms; *U* = 17, *p* = 0.1111).

### Discussion

The maturation of cortical circuits is coordinated by genetic and experience-dependent mechanisms [27]. The susceptibility of developing cortical circuits to environmental factors begins in the womb [28] and this is underscored the capacity of human newborns to immediately interact with their caregivers [7, 29, 30].

The last trimester of gestation is marked by rapid cortical growth [31, 32]. While the premature exposure to the extrauterine environment during this period may interfere with the maturation of association areas and increase the risk of neurodevelopmental impairment [33], the rate of cortical maturation is not synchronous across cortical regions [4, 34]. In the primary auditory cortex, for instance, developmental changes in cortical microstructure have largely occurred by 28 weeks of gestation [4]. This differential pattern of cortical maturation might explain why non-primary areas are more vulnerable to disruption due to premature exposure to the extrauterine environment [35, 36] while primary areas may experience an acceleration in maturation [9, 10]. According to our findings (see Fig. 1b), the earlier exposure to extrauterine sound stimulation in late-preterm infants probably speeds up the maturation of auditory cortical circuits and improves the efficiency of auditory input processing in this population during the first months of postnatal life [37].

An earlier study [38] had already reported that P1 latency was similar in 1-month-old terms and preterms. However, our study is the first to show that P1 latency in a group of 3 months old infants is shorter in moderate-to-late preterms (see Fig. 1b). Previous works had shown that P1 latency steadily decreases from around 250 ms in 1-month-old infants towards 100 ms in adults [39, 40]. The smaller P1 latency of preterms in the 3-month CA group probably reflects the accelerating maturational effects of early exposure to the extrauterine environment, which usually includes speech stimulation [41].

Other studies had already investigated the effect of preterm birth on the maturation of auditory cortical pathways in pre-schoolers using P1 as a biomarker [42–45]. However, the preterms in those studies were classified as extreme/very-preterm and the poor results they observed in comparison to controls may have been influenced by concurrent clinical conditions associated with extreme prematurity [45].

The neural mechanisms associated with the accelerating effects of preterm birth on the maturation of cortical pathways remain to be determined. One possibility is an increase in the effectiveness of thalamocortical connectivity with the primary auditory cortex [1] due to precocious exposure to the external social environment. A similar effect was reported previously in the primary somatosensory cortex of very preterm infants (GA < 33 weeks) following the premature exposure to activities such as breastfeeding and bottle-feeding [10].

A previous study [46] had shown that the latencies of components N1 and P2 are shorter in term than in preterm infants at 3 months of GA. While this result is the opposite we observed in the present work, we suppose this difference stems from the choice of auditory stimuli and the biomarker for physiological maturation. In that study [46], the stimulus was a click while we used speech stimuli (the phoneme/da/). Also, we used the latency of the P1 component as a biomarker, the gold standard for evaluating the maturation of cortical auditory pathways [17, 21, 23, 47, 48].

Our results are corroborated by other studies that show the advantages of prematurity in auditory recognition memory [49], binocular vision [9], and language comprehension [50]. Thus, even though preterm birth is associated with many neurodevelopmental risks, especially in small for gestational age (SGA) infants [32], the early exposure to socially relevant stimuli can enhance the maturation of sensory pathways [51]. Though our results differ from studies using visual evoked potentials (VEP) that show that preterm birth negatively affects the development of visual pathways [52, 53], the preterm group in those studies was composed of very preterm infants, which may have been SGA at birth.

### Conclusion

The present results reinforce the notion that early exposure to socially relevant environments contributes to the adaptive maturation of sensory pathways. This understanding is of practical importance since preterm birth is on the rise worldwide. Many preterms need to remain hospitalized in neonatal intensive care units (NICU), isolated from their parents, and subject to continuous loud noises or visual deprivation [54]. These conditions can be further detrimental to the maturation of cortical sensory circuits due to their effect on the levels of stress hormones. Fortunately, the young brain is remarkably resilient and can overcome early insults when provided access to appropriate care, stimulation, and follow-up measures [55].

## Limitations

The main limitations of the present work are (1) the small sample number, (2) the need to use a cross-sectional, instead of a longitudinal experimental design, and (3) the fact that we only considered the role of extrinsic variables (environmental exposure), while it is known that cortical maturation is influenced by intrinsic variables as well.

## Supplementary information

**Additional file 1: Table S1.** Correlation and linear regression values for P1 latencies.

## Data Availability

The data that support the findings of this study are available on request from the corresponding author, AP. The data are not publicly available due to ethical restrictions.

## Abbreviations

ABR: Auditory brainstem response
CA: Corrected age
CAEP: Cortical auditory evoked potential
DPOAE: Distortion Product Otoacoustic Emissions
GA: Gestational age
NICU: Neonatal intensive care unit
SGA: Small for gestational age
VEP: Visual evoked potential
