# Supplementary material for: Early exposure to environment sounds and the development of cortical auditory evoked potentials of preterm infants during the first 3 months of life
Source: BMC Res Notes. 2020 Jun 26;13:303. doi: 10.1186/s13104-020-05129-8 (PMC7318486; doi:10.1186/s13104-020-05129-8)
Supplement: Supplementary file 1 — Additional file 1: Table S1. Correlation and linear regression values for P1 latencies. [file 13104_2020_5129_MOESM1_ESM.docx]

**TABLE S1**: Correlation and linear regression values for P1 latencies

|  | *n* | | Pearson’s (r) | *P-value* | Slope | | Intercept |
| --- | --- | --- | --- | --- | --- | --- | --- |
| Peak Latency | |  | |  | |  | |
| P1 (1 month) | 11 | | 0.06 | 0.460 | 5.57 ± 7.21 | | 99.63 ± 262.30 |
| P1 (3 months) | 12 | | 0.32 | 0.056 | 9.56 ± 4.44 | | -144.30 ± 160.50 |
